# The Enigma of Tau Protein Aggregation: Mechanistic Insights and Future Challenges

**DOI:** 10.3390/ijms25094969

**Published:** 2024-05-02

**Authors:** Huiting Zheng, Huimin Sun, Qixu Cai, Hwan-Ching Tai

**Affiliations:** State Key Laboratory of Vaccines for Infectious Diseases, Xiang An Biomedicine Laboratory, State Key Laboratory of Molecular Vaccinology and Molecular Diagnostics, National Innovation Platform for Industry-Education Integration in Vaccine Research, School of Public Health, Xiamen University, Xiamen 361102, China

**Keywords:** amyloids, oligomers, fibrils, protein misfolding, phase separation, Alzheimer’s disease

## Abstract

Tau protein misfolding and aggregation are pathological hallmarks of Alzheimer’s disease and over twenty neurodegenerative disorders. However, the molecular mechanisms of tau aggregation in vivo remain incompletely understood. There are two types of tau aggregates in the brain: soluble aggregates (oligomers and protofibrils) and insoluble filaments (fibrils). Compared to filamentous aggregates, soluble aggregates are more toxic and exhibit prion-like transmission, providing seeds for templated misfolding. Curiously, in its native state, tau is a highly soluble, heat-stable protein that does not form fibrils by itself, not even when hyperphosphorylated. In vitro studies have found that negatively charged molecules such as heparin, RNA, or arachidonic acid are generally required to induce tau aggregation. Two recent breakthroughs have provided new insights into tau aggregation mechanisms. First, as an intrinsically disordered protein, tau is found to undergo liquid-liquid phase separation (LLPS) both in vitro and inside cells. Second, cryo-electron microscopy has revealed diverse fibrillar tau conformations associated with different neurodegenerative disorders. Nonetheless, only the fibrillar core is structurally resolved, and the remainder of the protein appears as a “fuzzy coat”. From this review, it appears that further studies are required (1) to clarify the role of LLPS in tau aggregation; (2) to unveil the structural features of soluble tau aggregates; (3) to understand the involvement of fuzzy coat regions in oligomer and fibril formation.

## 1. Tau Protein Function and Dysfunction

The microtubule-binding protein tau is an enigmatic protein. On one hand, fibrillar aggregates of tau protein are found in over twenty neurodegenerative disorders [1,2,3], which would seem to imply a strong propensity for misfolding and forming cross-β amyloid structures. On the other hand, native tau protein is an intrinsically disordered protein with exceptional solubility and heat stability [4,5,6], and neither wild-type tau nor hyperphosphorylated tau (p-tau) have been found to self-assemble into fibrils in vitro. Moreover, different tauopathies exhibit very diverse fibrillar conformations and neuropathological features [7,8,9]. The molecular mechanisms that induce tau misfolding and aggregation in neurodegenerative disorders remain largely unclear. In this review, we will discuss current knowledge about the aggregation mechanisms of tau protein and the critical gaps in our understanding.

### 1.1. Tau Isoforms and Domains

Tau protein is an important member of the microtubule-binding protein family, and it plays a critical role in regulating microtubule dynamics. In the brain, tau is mostly expressed in neurons, where it promotes microtubule assembly and stabilization, which are important for neuronal morphology and axonal transport [10,11,12]. In mature, healthy neurons, tau is mostly axonal, but a subpopulation is also found in dendrites [13], postsynaptic terminals [14], and extracellular vesicles [15,16]. Low levels of tau expression are also found in oligodendrocytes and astrocytes [17].

**Figure 1:**
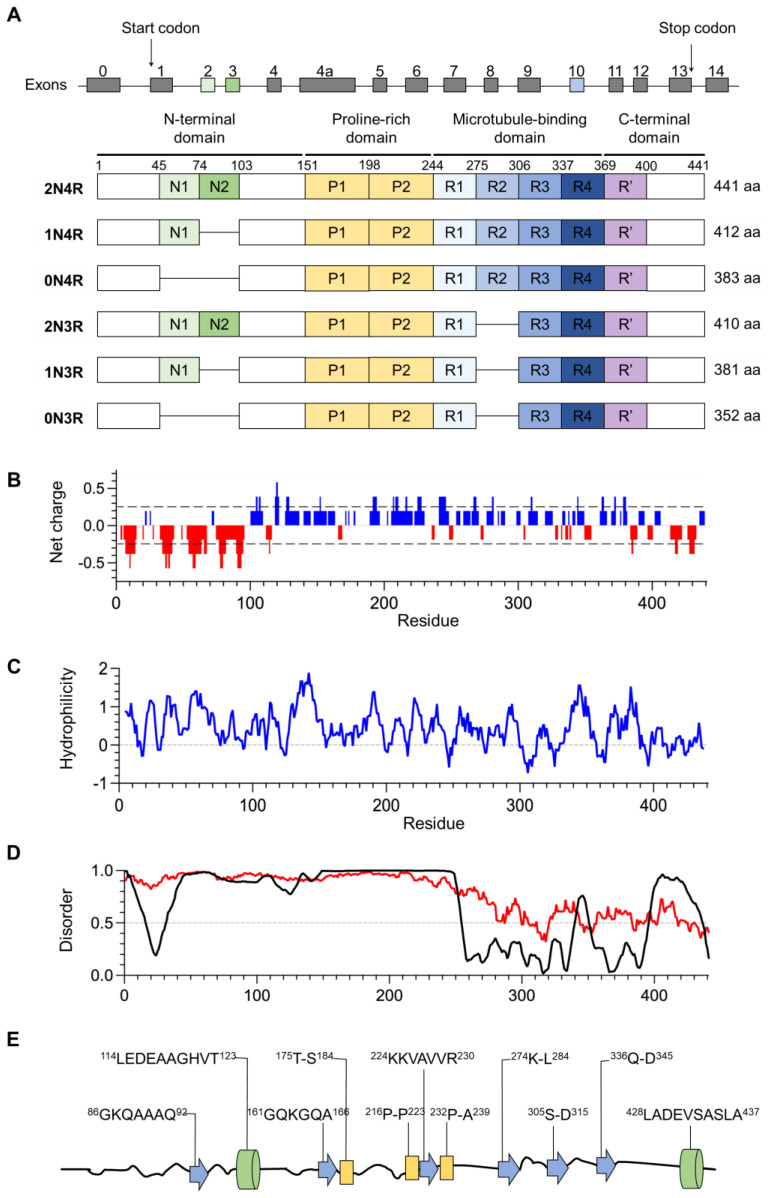
The splice variants of *MAPT* and the domain structures of tau protein. (**A**) Human brain tau has six major splicing isoforms. Exons 2, 3, and 10 are alternatively spliced. (**B**) Charge distribution along tau protein is calculated using CIDER [26]. (**C**) Hopp-Woods hydrophilicity scale calculated using Protscale (http://web.expasy.org/protscale/, accessed on 1 February 2024). (**D**) Disordered regions predicted by PONDR VLXT [27] (black curve) and IUPred3 [28] (red curve). (**E**) Structural motifs in native tau protein determined by solution NMR: α-helix (green), β-strand (blue), poly-proline helix (yellow) [5].

The tau gene (*MAPT*) is located on chromosome 17 and consists of 16 exons. The alternative splicing of *MAPT* is developmentally regulated, with six major tau isoforms expressed in the brain: 0N3R (352 aa), 1N3R, 2N3R, 0N4R, 1N4R, and 2N4R (441 aa) (Figure 1A) [18]. Here, N refers to the N-terminal regions encoded by exons 2 and 3. The 4R refers to four microtubule binding repeats (MTBRs, R1–R4), while the 3R tau is missing R2, encoded by exon 10. The physiological relevance of different tau isoforms is only partially understood [19]. 3R tau is associated with early development, but its affinity to microtubules is lower than that of 4R tau. The 2N inserts seem to promote somatodendritic retention [20]. In the peripheral nervous system, there is a longer tau isoform termed “big tau” with ~700 aa [21]. In this article, amino acid residues are labeled according to their positions in 2N4R full-length human tau.

Tau protein consists of four major regions: the N-terminal domain (NTD), the proline-rich domain (PRD), the microtubule-binding domain (MTBD), and the C-terminal domain (CTD). The domain structures, charge distributions, hydrophobic regions, disordered regions, and transient structural motifs of tau protein are illustrated in Figure 1. At neutral pH, NTD and CTD are negatively charged, while PRD and MTBD are positively charged. On the other hand, the surface of the microtubule is negatively charged. In addition to R1-R4, a fifth MTBR called R’ (369–399) has been identified by nuclear magnetic resonance (NMR) spectroscopy. R4 and R’ are tightly bound to microtubules, while R1-R3 are more flexibly bound, and the presence of P2 (198–243) also enhances binding [22]. The NTD is also called the projection domain (residues 1–150), which is not in contact with microtubules and tends to interact with bilayer membranes and membrane-associated proteins. The PRD (151–243) is a module for regulating signaling, and it interacts with kinases such as Fyn and Src. The abundance of Ser, Thr, and Pro residues also makes the PRD a target region for proline-directed kinases such as GSK3β and CDK5 [23,24]. The extreme N-terminus (2–18) is sometimes called the phosphatase activation domain (PAD), and its interaction with protein phosphatase 1 can regulate fast axonal transport [25].

### 1.2. Tau Inclusions in Neurodegenerative Disorders

The common features of many neurodegenerative disorders are the misfolded aggregates of one or a few critical proteins. Hence, they are also called protein misfolding disorders or protein conformational diseases. Misfolded proteins form inclusions called amyloids, which appear as nanosized fibrils/filaments with extensive cross-β structures [8,9,29,30]. The most common neurodegenerative disorder is AD, which accounts for ~60% of senile dementia cases and is expected to affect ~150 million people by 2050 [31]. AD is characterized by two protein lesions: senile plaques containing beta-amyloid (Aβ) peptide and neurofibrillary tangles (NFTs) containing tau [32,33]. In addition to NFTs (somatodendritic distribution), fibrillar tau lesions in AD are also found in dystrophic neurites (associated with neuritic plaques), neuropil threads (in distal axons and dendrites), and ghost tangles (left by vanished neurons) [32].

Since the immunohistochemical detection of tau protein associated with AD NFTs [34,35] and the confirmation of tau as the main component of the constituent paired helical filaments (PHFs) [36] nearly four decades ago, tau inclusions have been further identified in dozens of neurodegenerative disorders, more than any other amyloid protein. [1,2,3,37]. Different tauopathies display a wide range of pathological features with different types of tau inclusions affecting different brain regions and cell types. Based on the biochemical composition of inclusions, tauopathies may be classified into three categories: (1) 3R tauopathies, including Pick’s disease (PiD); (2) 4R tauopathies, including progressive supranuclear palsy (PSP), corticobasal degeneration (CBD), argyrophilic grain disease (AGD), globular glial tauopathy (GGT), and aging-related tau astrogliopathy (ARTAG); (3) Mixed 3R/4R tauopathies, including AD, chronic traumatic encephalopathy (CTE), primary age-related tauopathy (PART), and tangle-only dementia (TOD). In most tauopathies, the majority of tau inclusions are found within neurons. For AD and PART, tau inclusions occur only in neurons, forming pre-tangles (p-tau accumulation) and NFTs in the somatodendritic compartment. For ARTAG, tau inclusions are only found in astrocytes. For CTE, tau inclusions are both neuronal and astrocytic. For PiD, PSP, CBD, GGT, and AGD, tau inclusions are found in neurons, astrocytes, and oligodendrocytes [1,2,3,37].

Tau filaments have an ordered cross-β core that resides in the MTBD, and its β-sheet formation is thought to be initiated around two hexapeptide motifs at the beginning of R2 and R3 (V_275_QIINK_280_ and V_306_QIVYK_311_). Tau does not have extended hydrophobic regions (Figure 1B), and its aggregation does not seem to be driven by hydrophobic interactions [38,39]. Aggregated tau in the brain is hyperphosphorylated at dozens of Ser/Thr sites [40,41], which would render it even more hydrophilic (tau_441_ has 45 Ser and 35 Thr). It has been estimated that each human tau carries ~2 phosphates under physiological conditions, and fibrillar p-tau in AD carries ~8 phosphates [42]. Dozens of phosphosites have been identified on AD PHF tau, being concentrated in the PRD and CTD. The most important consequence of hyperphosphorylation is the dissociation of tau from negatively charged microtubules due to electrostatic repulsion, which is an initiation event for tau aggregation [43,44]. It has also been suggested that p-tau is associated with hypometabolism. Tau becomes hyperphosphorylated in hibernating mammals such as bears, Syrian hamsters, and arctic squirrels, which is reversed upon arousal [45]. So far, tangles have only been reported in aged bears but not in other mammals [46].

While mutations in *APP*, *PSEN1*, and *PSEN2* in the Aβ-generation pathway may lead to familial AD showing plaques and tangles [47], mutations in *MAPT* may lead to tangle formation but not plaques, resulting in frontotemporal dementia (FTD) or conditions similar to CBD, PSP, or PiD [37,48]. Expression of human tau P301L and P301S mutants (from familial FTD) are frequently used to generate transgenic murine models of tauopathy due to their misfolding propensities [49]. However, these are 4R tauopathy mouse models incapable of producing 3R/4R aggregates like those in human AD. Transgenic mouse tauopathy models and other related AD animal models have been reviewed elsewhere [49,50]. It is generally thought that tau hyperphosphorylation and aggregation damage neurons through gain-of-toxic-function rather than loss-of-normal-function, causing microtubule destabilization, axonal traffic disruption, altered neurotrophin signaling, synaptic dysfunction, and mitochondrial damage [51,52].

## 2. Aggregation Mechanisms of Tau

### 2.1. Native Tau Does Not Self-Assemble into Fibrils

In its native state, tau is a monomer and an intrinsically disordered protein (IDP) that lacks well-defined tertiary structures. It is highly soluble and heat-stable, maintaining its solubility even under boiling conditions [12,53]. Although native tau monomer is mostly in a random-coil state, some transient tertiary structures may still exist. Native tau shows a global fold that brings the N-terminus and C-terminus into close proximity with MBTD in a “paperclip” conformation [5,54]. The interaction between the C-terminus and MTBD has an inhibitory effect on tau aggregation. In the absence of such interaction, the extreme N-terminus comes into contact with R3 to initiate aggregation [55]. So far, none of the six tau isoforms have been reported to self-assemble into fibrils in the absence of cofactors or aggregate seeding [56,57]. Under certain conditions, 2N4R tau may self-assemble into low-n oligomers via interactions in its N-terminal half (residues 45–230) [58].

**Figure 2:**
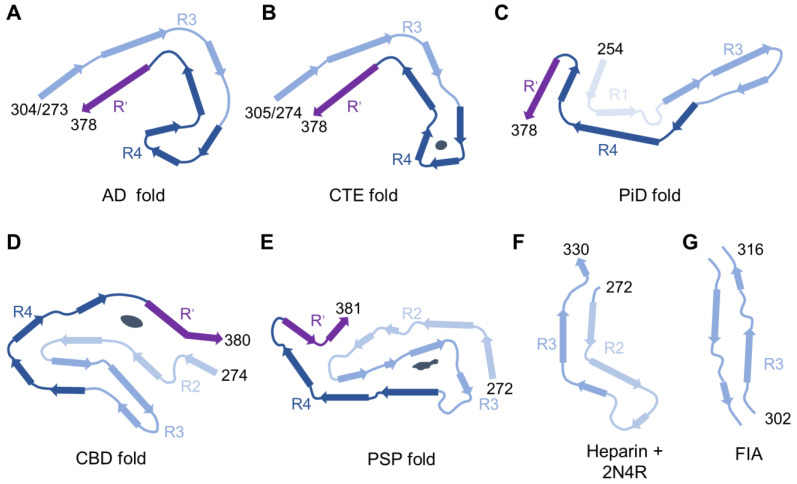
Tau fibril atomic structures resolved by cryo-EM. Misfolding structures from various tauopathies: (**A**) two-layered fold in AD for 3R + 4R tau; (**B**) two-layered fold in CTE for 3R + 4R tau; (**C**) Two-layered fold of 3R tau for PiD; (**D**) Four-layered fold of 4R tau in CBD; (**E**) Three-layered fold of 4R tau in PSP [7]. The black objects represent unidentified electron densities. For comparison, structures of in vitro aggregation products are shown: (**F**) recombinant 2N4R tau induced by heparin [66]; (**G**) First intermediate amyloid (FIA) fibril formed by dGAE fragments without cofactors [63].

Certain heavily truncated forms of tau are known to form fibrillar aggregates by themselves, such as the fragment containing aa 297–391 called dGAE. By itself, dGAE can form twisted, filamentous aggregates similar to PHFs in AD patients. By X-ray diffraction, dGAE filaments display cross-β structures commonly associated with protein amyloids [59,60,61]. With recent advances in cryo-electron microscopy (cryo-EM), the atomic structures of fibrillar core regions can be resolved (see Figure 2 for some examples), but the remaining regions of the tau protein still appear as an unresolved “fuzzy coat” [8]. Recent studies have shown that, under specific conditions, dGAE may form filaments structurally similar to those in AD (with MgCl_2_) or in CTE (with NaCl), depending on buffer conditions. With 2N4R tau, however, no aggregation is observed, which implies that both the N-terminus and C-terminus may inhibit fibrillation [62]. When dGAE aggregation is monitored at different time points, the earliest fibrillar aggregates observed by cryo-EM are called first intermediate amyloid (FIA) filaments (Figure 2G). It has a very small core region comprised of residues 302–316, which covers the R3 hexapeptide (306–311) which is considered as the nucleation center of tau fibrillization [63]. However, the aggregation process of dGAE (without granular oligomer intermediates) probably differs from that of full-length tau (with granular oligomer intermediates; see Section 3.3).

Even though tau filaments in human tauopathies are hyperphosphorylated, p-tau does not self-assemble into filaments in vitro. For instance, 2N4R tau expressed in insect cells may carry 12–20 phosphates per polypeptide, but their self-assembly products are mostly oligomers and occasionally a few fibrils [64]. When 0N4R tau is hyperphosphorylated by kinases in vitro, it spontaneously forms amorphous aggregates but not filaments [65]. On the other hand, when all six human tau isoforms are hyperphosphorylated using rat brain extracts, the spontaneous formation of filaments is observed when the stoichiometry reaches 10–15 phosphates per polypeptide. It implies that filament formation in vivo may require both hyperphosphorylation and additional cofactors [57].

### 2.2. Inducers of Tau Aggregation In Vitro

Our mechanistic understanding of tau aggregation has mostly come from in vitro studies. Various negatively charged cofactors are known to induce wild-type tau aggregation, including polyanions like DNA [67], RNA [68], heparin [69], polyglutamate [70], or polyphosphates [71]; anionic micelles formed by arachidonic acid or 9-palmitoleic acid [72]; and aromatic anionic dyes such as thiazine red [73], Congo red [74], and thioflavin S [75]. It is generally thought that anionic inducers could disrupt long-range electrostatic interactions in tau to promote aggregation, but the molecular details remain little understood.

Among various aggregation inducers, heparin is the most commonly utilized for in vitro studies for two reasons. First, heparin is structurally similar to sulfated glycosaminoglycans (GAG) that have been found to colocalize with tau inclusions in AD tissues [69]. Second, heparin induction is usually robust and reproducible. GAGs such as heparan sulfate and chondroitin sulfate are post-translational modifications (PTMs) found in a class of glycoproteins called proteoglycans. GAGs are either found on secreted proteins or the extracellular side of membrane proteins, and the reason for their colocalization with intracellular tau inclusions remains unclear. It has been proposed that extracellular tau binds to proteoglycans extracellularly, forms seeding aggregates, and becomes internalized together [76]. NMR studies have shown that heparin induces the formation of β-strands and α-helices in tau, interacting with the hexapeptide motifs (275–280 and 306–311) [39,77]. Although heparin-induced 2N4R tau filaments show paired helical morphologies resembling AD PHFs, their atomic structures are quite distinct [66,78] (Figure 2). It has been reported that tau fibrils induced by heparin still require bound heparin molecules for their seeding capabilities, and ditto for RNA induction [79].

By single-molecule mass photometry, native 0N4R tau has been shown to exist in equilibrium among monomers (major), dimers, and trimers [80]. This is consistent with the formation of tau dimers and trimers at the microtubule surface, mediated by electrostatic interactions in the N-terminal half of tau [81,82]. When Congo red is added to induce aggregation, mass photometry reveals oligomer formation (6 to 25-mer) with lag-phase kinetics that is consistent with a linear templated conversion model (1 → 2 → 3 → 4 → … → N). In this model, monomers dissociated from oligomers are misfolded, while both native and misfolded monomers may add to existing oligomers [80]. It has also been reported that tau monomers dissociated from preexisting aggregates (from cells and brains) may carry misfolded conformations [83,84,85]. Thus, the aggregation process of tau appears to share the general principles of other amyloids, involving nucleated growth and templated misfolding [29,86].

**Figure 3:**
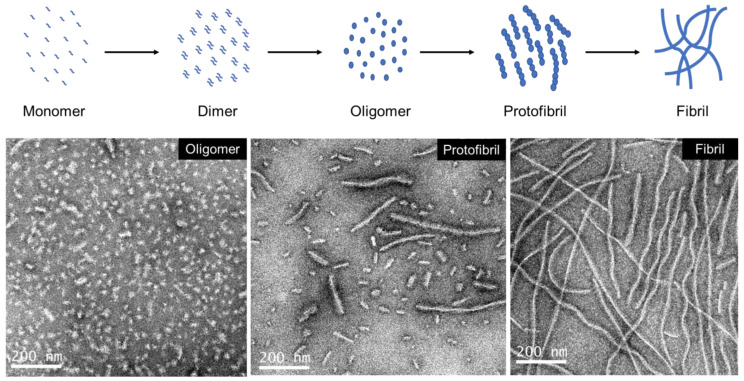
The different stages of tau aggregation. In this simplified model, tau aggregation proceeds through different stages—monomers, dimers, oligomers, protofibrils, and fibrils. TEM images of heparin-induced 2N4R tau aggregates are shown at the bottom. These oligomers (high-n) and protofibrils are intermediate products purified by SEC. The fibrils are the final products of extended reactions.

In general, tau aggregation proceeds through multiple stages: monomers (native) → dimers (misfolded) → oligomers (granules) → protofibrils (short rods) → fibrils/filaments (insoluble threads) [87,88,89,90] (Figure 3). Here, dimers and low-n oligomers may be considered prenucleation clusters, while high-n oligomers are nuclei for fibril growth. The formation of oligomers is considered primary nucleation, while secondary nucleation may occur when fragments break off from fibrils and serve as seeds for templated misfolding [91]. The nomenclature of tau aggregates based on size and shape is loosely defined, without a general consensus. Here, we consider granules with diameters of 4–40 nm as oligomers, short rods with lengths of 40–400 nm as protofibrils (also called short fibrils or short filaments), and fibers longer than 400 nm as filaments/fibrils. These three species may be semi-purified based on density differences using sucrose gradient centrifugation [89,92]. In our experience, a more stringent separation of protofibrils, high-n oligomers, and low-n oligomers may be achieved by size exclusion chromatography (SEC) using large-pore resins (~200 nm). High-n oligomers (>20) are also called high-molecular-weight tau aggregates. Transmission electron microscopy (TEM) images of fibrils (recombinant tau induced by heparin), SEC-purified protofibrils, and SEC-purified high-n oligomers are shown in Figure 3.

### 2.3. Tau Aggregation In Vivo

**Figure 4:**
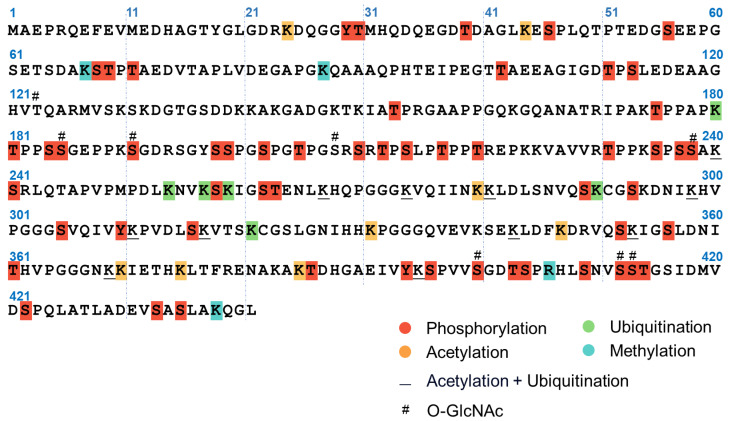
Tau PTM distributions. The amino acid sequence of 2N4R tau is labeled with sites of O-GlcNAc glycosylation [95], phosphorylation, acetylation, ubiquitination, and methylation [94].

Tau is known to be regulated by a wide variety of PTMs, including truncation, phosphorylation, O-GlcNAc glycosylation, acetylation, methylation, ubiquitination, etc. [93,94,95] (Figure 4). Aggregated tau from AD brains is hyperphosphorylated [42,96], imparting negative charges to dissociate tau from the negatively-charged microtubules, which appears to be a prerequisite for misfolding [97,98]. Tau contains dozens of phosphosites targeted by multiple kinases such as GSK3β, CKD5, p38α MAPK, PKA, ERK, JNK, FYN, etc. [44,99,100]. On the other hand, protein phosphatase 2A (PP2A) has been shown to interact both with microtubules and tau, and its activity appears to be 50% lower in AD brains [101]. Therefore, the combined action of kinase activation and phosphatase inhibition may contribute to tau hyperphosphorylation and misfolding.

AD NFTs contain all six tau isoforms [18], which appear to be incorporated as intact full-length proteins [102]. In the pre-tangle stage, the extreme N-terminus is folded onto the R3, creating a conformer recognizable by Alz-50 and MC-1 antibodies [103,104]. During tangle maturation, C-terminal truncation occurs gradually, followed by N-terminal truncation [102,104]. The majority of sarkosyl-insoluble fibrillar tau have apparent molecular weights around 40–45 kDa, being truncated in the CTD. One of the major proteolytic sites in the CTD is after D421, cleaved by various caspases. In addition, a wide variety of truncated fragments, down to ~15 kDa, are found in AD brains. Proteolytic sites have been found in all four major domains of tau, with over thirty fragments identified so far [105,106,107]. In general, tau fragments containing MBTD are more prone to aggregation than untruncated tau [108,109]. Recent studies have also found N-terminal truncations in oligomer and protofibril species from AD brains [110,111].

Although there is a strong correlation between tau pathology and certain PTMs [94], it is difficult to determine which PTM sites are necessary or sufficient for tau misfolding in vivo. Under different contexts, different phosphosites have been suggested as pathological correlates. In AD PHF tau, the following sites are often considered to be pathologically relevant: Y18, T181, S198/S199/S202/S205, T212/S214/T217, T231/S235, S262, S396/S400/S404, S416, and S422 [112,113]; In APP/PS1 mouse synaptic sites, the early hyperphosphorylation sites appear to be 181, 198/199/202, 231, 396/400/404, and 416 [114]. In non-demented elderly, phosphorylation often occurs at 181, 198/199/202/205, 212/214/217, 231/235, 262, and 396/400/403/404 [94]—it is unclear if these reflect normal physiology or aging-related changes. Considering only phosphorylation, truncation, and alternative splicing, there could be hundreds of tau proteoforms, so it is very difficult to determine which species represent the critical seeding species, or how they become involved in templated misfolding in vivo. Despite the high sensitivity of shotgun mass spectrometry in detecting phosphosites, it could neither reveal proteoforms (because peptides from different proteoforms are mixed together) nor quantify the stoichiometry at individual phosphosites (due to the clustering of phosphosites) [94,114]. Hence, phospho-specific antibodies are often used for the relative quantification of phosphosite changes in biological samples, but their specificity needs to be carefully validated, especially around clustered phosphosites [112].

### 2.4. Structural Diversity of Tau Fibrils in Different Tauopathies

A recent breakthrough in understanding tau misfolding has come from the atomic structures of fibrillar cores resolved by cryo-EM, revealing different folds associated with different tauopathies (Figure 2). An important lesson from cryo-EM studies is that fibril morphologies under TEM are not correlated with atomic structures. For instance, two fibrillar morphologies are found in AD—PHFs and straight filaments—but their core atomic structures turned out to be highly similar. The AD filament core contains two “C-shape” structures spanning R3, R4, and R’ (G273-E380 of 3R tau and G304-E380 of 4R tau) [115,116]. The AD tau fold is also found in PART, familial British dementia, familial Danish dementia, and certain tauopathies caused by prion protein mutations [7,117].

In CTE, the ordered core corresponds to K274-R379 in 3R tau or S305-R379 in 4R tau, also forming a “C-shape” fold [118]. Moreover, in Guam amyotrophic lateral sclerosis (ALS)/parkinsonism-dementia complex and subacute sclerosing panencephalitis (following measles infection), tau filaments also adopt the CTE fold, implying that neuroinflammation may be the underlying cause [119,120]. On the other hand, 3R tau filaments in PiD have a “J-shape” fold spanning K254-F378 (R1, R3, and R4) [121]. Both C- and J-shape folds are two-layered structures. In CBD, there are two types of 4R tau filaments with four-layered folds that correspond to K274-E380 (R2 to R4), enclosing a non-proteinaceous density [122]. For AGD and ARTAG, the folds are similar to those of CBD. For PSP and GGT, they have similar three-layered folds [7]. Altogether, over a dozen different folds have been identified from various tauopathies [7,9], while many additional folds have been found with recombinant tau fibrils prepared under different conditions [62,66]. It implies that tau fibrils are not thermodynamically favored products but kinetically determined products. In cryo-EM structures, there are sometimes unidentified electron densities that may present cofactors. Moreover, a large proportion of tau protein still belongs to the unresolvable “fuzzy coat”. It remains challenging to understand how cofactors and fuzzy-coat regions affect the kinetic pathways of misfolding.

## 3. Soluble Tau Aggregates—Oligomers and Protofibrils

### 3.1. Discovery of Tau Oligomers

While early tau research was focused on fibril formation and deposition, increasing attention has turned to soluble misfolding intermediates (oligomers and protofibrils), trying to understand their assembly, structure, toxicity, and propagation [123]. In AD tissues, tau oligomers were discovered by immunoprecipitation (using C-terminal tau antibody) from brain extracts, appearing as granules under TEM with ~20 nm in diameter, corresponding to ~40 p-tau subunits. By circular dichroism, granular oligomers exhibit a lower percentage of β-sheet than fibrils and a lower percentage of random coils than monomers [89,124]. In another study, immunoprecipitated (using T22 antibody) tau oligomers from AD brains are 4–8 nm in diameter, appearing mostly as trimers [125]. The reported size differences may be due to differences in capture antibodies, elution conditions, or purification procedures. Tau oligomers have been identified inside the synaptic terminals of AD tissues, and their accumulation correlates with the disruption of the ubiquitin-proteasome system [14]. Under TEM, no fibrillar tau has been observed inside synaptic terminals, and the size and shape of synaptic tau oligomers remain unknown. They appear to be hyperphosphorylated and recognizable by conformational antibodies such as Alz-50 and T22 [126,127]. Tau oligomers, both recombinant and AD-derived, could disrupt long-term potentiation when applied extracellularly to hippocampal slices [128].

On the other hand, the presence of protofibrils in AD tissues is harder to ascertain. In the sarkosyl-insoluble tau fraction of AD tissues, protofibrils (short fibrils) and long fibrils are detected, but it remains unclear if protofibrils are fragmentation products of long fibrils during homogenization and purification [129]. It has been proposed that pre-tangles may contain short tau fibrils [130], but others consider pre-tangles as oligomeric or amorphous aggregates of p-tau (marked by the AT8 epitope pS202/pS205) [131]. Cryo-EM studies have recently identified short tau fibrils inside extracellular vesicles isolated from AD tissues [132]. With recombinant tau and heparin, it has been proposed that granular oligomers can form bead-on-a-string curvilinear structures that gradually evolve into short rods with smooth surfaces [88]. Whether this protofibril formation process also occurs in vivo remains unclear.

### 3.2. Tauopathy Propagation and Prion-like Mechanisms

In neurodegenerative prion disorders, misfolded aggregates of prion protein (PrP) have been shown to self-replicate and self-propagate in the brain, transmitting between humans and across different mammalian species [133]. While non-prion amyloids appear to be non-infectious, there is increasing evidence to suggest that they can self-propagate within the central nervous system through “prion-like” seeding mechanisms. Therefore, misfolded protein aggregates and “prion-like” transmission have become two unifying concepts used to explain various neurodegenerative disorders [30,134,135].

NFT deposition in AD exhibits a stereotypical spatiotemporal spreading pattern known as Braak Stages, which follows major axonal projection pathways [136,137,138]. There is growing evidence to suggest that soluble tau aggregates act as seeds that underlie this spreading [139,140,141]. Firstly, at a given brain region of AD subjects, soluble tau seeds are detected long before pre-tangles and tangles [142,143], which is also observed for tauopathy mice [144,145,146]. Secondly, p-tau oligomers (often truncated) are released by the synaptic terminals of AD patients [147,148]. Thirdly, the synaptic distributions of tau oligomers in AD and PSP tissues implicate their transmission from presynaptic to postsynaptic terminals [126,149]. Trans-synaptic transmission of tauopathy has also been demonstrated in animal tauopathy models [150,151].

Compared to insoluble filaments, soluble tau oligomers are more toxic, more seeding-capable, and more transmissive [152,153]. In cellular toxicity assays, recombinant tau oligomers are more toxic than corresponding fibrils [154]. When heparin-induced tau fibrils are vigorously sonicated, they are reduced to granular oligomers and show greater structural disorder (by circular dichroism), greater cellular toxicity, and greater seeding capacity [155]. Comparing oligomers and fibrils isolated from AD brains, the former is more toxic [156] and more capable of propagating tauopathy when injected into mice brains [110,125]. It is further shown that high-molecular-weight tau aggregates (>20-mer) but not low-n oligomers (2 to 8-mer) from AD brains are internalized by cultured neurons and propagate tau misfolding trans-synaptically [157]. Similarly, oligomers and protofibrils isolated from tauopathy mice, but not fibrils, are internalized by cultured neurons [158]. In rTg4510 mice, turning off P301L tau expression leads to cognitive improvement, but tangle deposition continues, implying that tangle formation is not damaging but may even help reduce toxic soluble aggregates [159]. The internalization of AD-derived tau oligomers by cultured neurons may depend on micropinocytosis mediated by heparan sulfate proteoglycans [160].

There is also evidence to suggest that Aβ oligomers may induce tau oligomer deposition at synapses. In mouse AD models, Aβ oligomers induce p-tau accumulation at synapses [114,161] and accelerate the spreading of tau pathology [162], but the synaptotoxic effects of Aβ are ameliorated by the genetic removal of the *MAPT* gene [163,164]. At AD-affected human synapses, the buildup of Aβ oligomers precedes that of tau oligomers [147]. When AD-derived tau oligomers are injected into the entorhinal cortex of macaque monkeys, tau pathology (neuropil threads and pre-tangles) spreads into connected brain regions in a manner consistent with Braak staging. Co-injection with Aβ oligomers further induces tangle deposition and synapse degeneration [165].

Although tau was originally identified as an intracellular protein, studies in animal and cellular models have shown that physiological tau is secreted through synaptic release [166], ectosomes [16], and exosomes [167], also found within tunneling nanotubes [168]. The propagation of tau aggregates may also occur through the above mechanisms. The majority of extracellular tau (physiological or pathological) appears to be vesicle-free, and a minor fraction is vesicle-associated [169,170,171]. Under physiological states, tau protein has been detected in the cerebrospinal fluid (CSF) of humans (truncated forms) and the CSF/interstitial fluid of mice [172,173], but the physiological function of extracellular tau remains unclear. In human AD subjects and tauopathy mice, tau oligomeric seeds (>20-mer) have been detected in the CSF [174].

### 3.3. Preparation and Detection of Tau Oligomers

Tau oligomers are metastable species that are challenging to prepare in vitro and difficult to detect in vivo. Table 1 summarizes various methods developed for generating oligomers from wild-type tau. With photo-crosslinking [175], H_2_O_2_ [129], oligomeric Aβ, or oligomeric α-synuclein [154] as inducers, dimers/trimers may be obtained. With heparin [89,176,177] or arachidonic acid [92,178] as inducers, larger oligomers with heterogeneous sizes may be obtained (around 20–100 subunits), but they are intermediate species that gradually convert into fibrils with longer reaction times. With current technologies, it has not been possible to probe the atomic structures of either large or small tau oligomers.

The detection of tau oligomers inside cells and tissues is challenging because they lack distinguishable morphologies under TEM and cannot be uniquely identified by pan-tau or phospho-tau antibodies. To detect tau oligomers, several conformation-specific antibodies have been generated against tau dimers and trimers. TOC1 (mouse monoclonal) was generated against photo-crosslinked dimers [175]. T22 (rabbit polyclonal) [179] and TOMA (mouse monoclonal) [180] were generated against oligomeric-Aβ-induced tau trimers. On the other hand, Alz-50 has also been shown to be reactive against AD synaptic tau oligomers [127], and MC-1 against AD brain-derived oligomers [89], although they supposedly recognize conformational changes leading to fibril formation [103]. The epitopes of TOC1 [181] and TOMA [182] are mapped to the PRD, which may be a region important for oligomer formation.

**Table 1:** Preparation of oligomeric aggregates from wild-type tau.

Monomer	Inducer	Purification	Size	Reference
His_6_-0N3R, His_6_-0N4R	arachidonic acid	none (do not form fibrils)	20–80 nm	King et al., 2002 [178]
2N4R	heparin	sucrose gradient	15–25 nm (~40-mer)	Maeda et al., 2007 [89]
2N4R	oligomers of Aβ or α-synuclein	SEC	trimer	Lasagna-Reeves et al., 2010 [154]
2N4R	heparin	SEC	~14 nm (40-mer)	Flach et al., 2012 [177]
2N4R	photo-crosslinker	gel electrophoresis	dimer	Patterson et al., 2011 [183]
His_6_-2N4R	arachidonic acid	sucrose gradient	6–16 nm	Combs et al., 2017 [92]
His_6_-2N4R	H_2_O_2_	SEC	dimer/trimer	Fa et al., 2016 [129]
2N4R	heparin (stabilized by glutaraldehyde)	SEC	5–50 nm	Das et al., 2020 [176]
p-tau from insect cell	none	none	5–30 nm	Tepper et al., 2014 [64]

A recent study examining the specificity of multiple α-synuclein conformational antibodies has found that so-called “oligomer-selective” antibodies also cross-react with fibrils [184]. Although TOC1, T22, and TOMA are conformational antibodies with low affinities against tau monomers, whether they also recognize protofibrils and fibrils has not been systematically investigated. Therefore, the detection of tau oligomers using conformational antibodies should be interpreted with caution, especially for immunostaining experiments. Heparin-induced aggregates are likely conformationally heterogeneous, but it remains difficult to characterize conformational diversities among subunits within a single aggregate entity or between aggregates of different types and origins.

## 4. Liquid-Liquid Phase Separation

Advances in genome sequencing and structural biology have revealed that many proteins contain domains without well-defined structures, called intrinsically disordered regions (IDRs), which often carry low sequence complexity. When the entire protein appears disordered, as in the case of tau, it is called an IDP. There is increasing evidence to suggest that IDRs and IDPs could mediate important cellular functions through macromolecular condensation, forming liquid-like or gel-like structures. Acting in concert with stereospecific macromolecular interactions, condensation through IDRs and IDPs may contribute to the formation of membrane-less organelles such as centrosomes, stress granules, and P-bodies [185,186,187].

In vitro, IDPs or IDRs often form condensates under high concentrations and molecular crowding conditions. The condensation process may go through several stages: free solute → liquid droplet → hydrogel/glass → solid. The formation of liquid-like droplets is also called coacervation or liquid-liquid phase separation (LLPS), mediated by transient, multivalent, low-affinity adhesive interactions. After hours or days, the droplets may undergo “maturation” or “aging” and harden into gel-like or glassy condensates [91,187,188,189]. The driving forces for condensation are complex, involving dipole-dipole, π-π, cation-π, electrostatic, hydrophobic interactions, hydrogen bonds, and/or transient cross-β structures [190,191]. LLPS may eventually lead to the formation of cross-β amyloid structures, which can either be physiological (functional amyloids) or pathological [186,191,192]. There is a growing interest in understanding whether tau aggregation is also associated with LLPS.

### 4.1. Tau LLPS In Vitro

So far, LLPS has not been observed with wild-type tau protein even at high concentrations, but many conditions for inducing LLPS have been reported, as summarized in Table 2. Because different forms of tau have been employed (truncated, mutated, GFP-fused, polyhistidine-tagged, etc.) and the protein purity may vary, caution is required before drawing generalized conclusions from in vitro LLPS experiments. The most common coacervation conditions involve molecular crowding by adding about 10% polyethylene glycol (PEG), 10% dextran, or 12.5% Ficoll [193,194,195]. With molecular crowding, LLPS is generally favored at physiological or low-salt concentrations, but disfavored at high-salt conditions, implying that electrostatic interactions may partly contribute to adhesive interactions. The low-salt condition also promotes the self-coacervation of a mutant 2N4R tau (C291S/C322S to eliminate all cysteines) in the absence of molecular crowding. At very high salt concentrations (4.75 M NaCl), His_6_-tau_2N4R_ (C291S/C322S) can undergo LLPS due to a “salting out” effect, which implies the involvement of hydrophobic interactions [196].

Tau is a protein with limited hydrophobicity and tau liquid droplets are not particularly sensitive to 1,6-hexanediol, which implies that hydrophobic interaction and cross-β structures have limited roles in LLPS. Moreover, tau is lacking in aromatic residues (0 Trp, 5 Tyr, and 3 Phe), so π-π and cation-π interactions are probably less important for phase separation. Both the N-terminal and the C-terminal half of tau can undergo LLPS under molecular crowding [194,197]. As the NTD is negatively charged and the PRD is positively charged, electrostatic interaction may play a role in the coacervation of the N-terminal half. The C-terminal half is capable of forming cross-β structures in addition to electrostatic interactions. Further research will be required to understand the driving forces in homotypic tau LLPS [206,207,208].

In addition to homotypic self-coacervation, tau LLPS involving heterotypic interactions is also observed. Polyanions such as RNA [197] or heparin [199,202], which are known to induce tau aggregation, can also induce tau LLPS. Moreover, adding the RNA-binding protein TIA1 and low amounts of RNA can promote tau LLPS [204]. Interestingly, 2N4R tau purified by heparin-sepharose has been reported to undergo LLPS at a low concentration (2.5 µM) in low-salt buffer without induction agents [199]. This reminds of a recent report that surface-immobilized heparin is sufficient to induce fibril formation when incubated with 2N4R tau solution (10 μM) [209]. It is plausible that immobilized heparin may induce conformation changes and/or oligomerization, promoting either LLPS or amyloid formation, but heparin leakage should also be carefully checked in such experiments.

Hyperphosphorylated tau expressed in insect cells and aggregation-promoting mutants (A152T, ΔK280, P301L, and P301S) still require molecular crowding to promote LLPS. Tau oligomers isolated from AD brains can also undergo LLPS with molecular crowding [194]. On the other hand, in vitro acetylation of 2N4R tau by p300 greatly impedes coacervation under molecular crowding [199]. Interestingly, liquid droplets (in 10% dextran) formed by hyperphosphorylated tau_2N4R_-EGFP-His_6_ can recruit tubulin dimers (α/β) and promote their polymerization into extended microtubules [195]. In comparison, AD PHF p-tau inhibits microtubule assembly even in the presence of normal tau [210]. Whether tau LLPS may promote microtubule assembly inside cells will require further investigation.

### 4.2. Tau LLPS Inside Cells

While it is relatively straightforward to demonstrate LLPS with purified proteins in test tubes, it is much harder to ascertain LLPS occurring inside live cells. Common criteria for detecting LLPS inside cells include roundness, droplet fusion, sensitivity to 1,6-hexanediol, and fluorescence recovery after photobleaching (FRAP). However, meeting these criteria cannot unequivocally establish LLPS phenomena inside cells [211]. Bearing these limitations in mind, several studies have suggested that tau proteins may form liquid-like droplets inside cells. When GFP-tau_441_ is expressed in primary neurons, they form droplet-like structures that show long-distance movements and have FRAP characteristics consistent with LLPS [194]. When 2N4R, 1N4R, and 0N4R-tau-EGFP are expressed in the HT22 neuronal cell line, droplet-like structures are abundant with 2N4R, less common with 1N4R, and absent with 0N4R tau. Moreover, 2N4R tau droplets colocalize with p62/SQSTM-1 protein [212]. It is also known that p62 can undergo LLPS, which is thought to promote proteasomal and autophagic degradation of proteolytic substrates [213]. It is plausible that N-terminal inserts may regulate tau protein degradation through coacervation.

When EGFP-0N4R_P301L_ tau is stably expressed in HEK293 cells, it uniformly fills the cell. Upon seeding with aggregates from P301L tauopathy mice, tau droplets are formed and show fusion and FRAP characteristics consistent with LLPS. Interestingly, such droplets may even transmit into neighboring cells through tunneling nanotubes. These droplets of mutant tau are moderately toxic to cells, accumulating around the nucleus and impairing nuclear transport. Thioflavin S may appear inside the larger droplets, suggesting the gradual formation of cross-β structures [214]. In another study, 2N4R tau is fused to Cry2 (a photoactivable protein) and mCherry (a fluorescent protein) to construct an optogenetic LLPS system. Upon blue light activation, Cry2 by itself can oligomerize but not phase separate. Upon photoactivation, Cry2-mCherry-tau inside the SH-SY5Y neuroblastoma cell line forms condensates associated with microtubules, colocalizing with EB-1 protein that binds to the end of microtubules. By testing various truncation mutants, it is determined that PRD mediates tau LLPS in this system [215]. In summary, multiple tau constructs and cell types have been used to demonstrate tau LLPS within the cellular environment. Together with ample evidence of tau coacervation in vitro, it is likely LLPS is an evolutionary feature of tau protein, and its physiological role may be related to microtubule regulation.

### 4.3. The Role of LLPS in Tau Aggregation

Insights into the relationship between LLPS and amyloid fibrils have initially come from the study of FUS and TDP-43, two RNA-binding proteins that form inclusions in FTD and ALS. They both contain an IDR that is responsible for oligomerization, phase separation, hydrogel formation (via cross-β structures), and fibrillar aggregation [216,217]. Concerning tau protein, however, the relationship between LLPS and fibril formation is less clear [206,207].

With p-tau_441_ in PEG solution, liquid droplets gradually harden into hydrogels and become thioflavin S-positive, reflecting the formation of cross-β structures, but no fibrils are observed [194]. With non-phosphorylated tau_441_-His_6_ and its P301L and S199E-S202E-T205E variants, the maturation of droplets did not lead to thioflavin T fluorescence, but conformational changes could be detected using oligomer-selective TOC1 antibody and PAD-conformation-sensitive TNT2 antibody [201]. With 0N4R tau in PEG-induced liquid droplets, no aggregation is detected. By adding RNA or RNA + TIA1, oligomer formation is detected by native gel electrophoresis and TOC1, but no thioflavin S-positive species or fibrils are found [204]. With His_6_-tau_255–441_(C291S/C322S), LLPS is induced by heparin and fibril formation is obvious after 16 h. Although fibrils appear to co-localize with tau droplets under the microscope, further examination revealed that heterotypic LLPS and fibrillation are independent processes that can occur under overlapping conditions [202].

When K18 tau (244–372, 100 μM) undergoes coacervation in 7.5% PEG, no further aggregation is observed after extended incubation. When 25 μM heparin is added to K18 in PEG, large liquid droplets are observed at 24 h, which condense into amyloid fibrils at 72 h [198]. On the other hand, when His_6_-Δtau187 (255–441) is incubated with RNA, the thioflavin T signal gradually increases over time (15 h) but no fibrils are observed [197]. Also, with His_6_-tau_2N4R_ (C291S-C322S) under high salt (4.75 M), liquid droplets of tau gradually become dehydrated and amyloid fibrils are formed inside. Although the high-salt condition is probably less physiologically relevant, it implicates dehydration as an important step in droplet maturation, which may further reduce intermolecular distances to promote higher-order self-assembly [196].

Taken together, it appears that wild-type tau does not spontaneously assemble into fibrils inside liquid droplets under molecular crowding, despite very high local concentrations. As these tau droplets mature, the formation of cross-β structures and oligomers may occur. It remains to be seen if such oligomers may act as seeds to promote fibrillation when they exit the condensates. Polyanion cofactors such as heparin could promote both LLPS and fibril formation, but whether nucleation and fibril growth occur outside or inside liquid droplets need to be carefully distinguished. The relationship between tau LLPS and fibrillation is complex and remains little understood, and it is particularly difficult to probe within the complex cellular milieu [192,206].

## 5. Tau Therapeutics

Over the last four decades, much has been learned about the biochemistry and neuropathology of tau protein, and there is also significant progress in tau translational research. In terms of tauopathy diagnosis, positron emission tomography (PET) tracers of tau fibrils [218,219] and blood biomarkers of tau [220,221] have proven clinically useful. In terms of tau therapeutics, more than thirty therapeutic agents have entered human clinical trials but none has received clinical approval yet. Clinical trials with tau-targeting therapies have been reviewed in detail [140,222,223,224,225], and updated information may be found on the alzforum.org and clinicaltrials.gov websites. Here, we will briefly summarize different therapeutic approaches to target tau pathologies.

Because pathological tau is hyperphosphorylated, there have been clinical trials with kinase inhibitors against GSK3β (Tideglusib) and Fyn (Saracatinib). An alternative approach is to enhance O-GlcNAc glycosylation to reduce phosphorylation, using inhibitors of O-GlcNAcase (LY3372689, BIIB113, ASN51, and ASN90). However, GSK3β, Fyn, and O-GlcNAcase are promiscuous enzymes not specific to tau pathways, so their inhibition may cause potential side effects. Another class of small-molecule drugs being clinically tested is based on the “molecular chaperone” idea, trying to prevent or modulate tau misfolding pathways to reduce toxicity, including methylene blue, HMTM (a methylene blue derivative), ACI-3024, and OLX-07010. In terms of biologic drugs, two antisense oligonucleotides against *MAPT* (NI0752 and BIIB080) have entered clinical trials, aiming to reduce tau expression via ribonuclease H-dependent mRNA degradation. Such therapies will likely also reduce physiological tau expression in non-degenerating brain regions.

**Figure 5:**
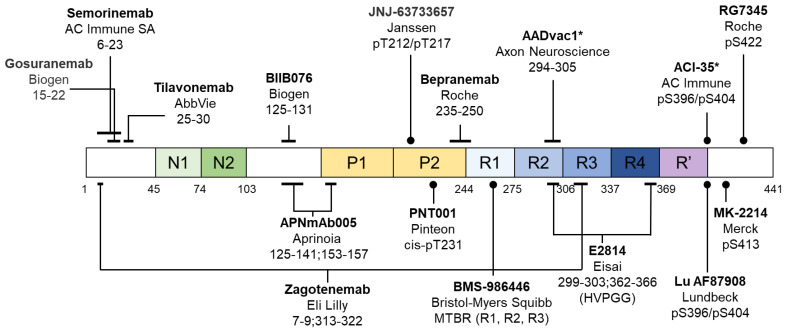
Epitope map of tau immunotherapies. Sixteen tau immunotherapies have entered human clinical trials, and their reported epitopes are shown, with * denoting active immunotherapies. See Table 3 for details.

There are two active tau immunotherapies (using peptide immunogens) and fourteen passive immunotherapies (by antibody injection) that have entered human clinical trials [140,222,223,224,225], summarized in Table 3. Their respective epitopes are illustrated in Figure 5. Nine immunotherapies are targeting linear sequences on tau (pan-tau antibodies), unlikely to differentiate between physiological and pathological tau species. Four of them target hyperphosphorylation sites (pT212/pT217, pT231, or pS396/pS404) associated with AD-PHF tau, but these sites are also phosphorylated in non-demented elderly [94]. Whether targeting these phosphosites might interfere with physiological tau function remains unclear. Two of them are conformation-specific antibodies—one selective for fibrils (Zagotenemab, humanized MC-1) [103] and one selective for soluble aggregates (APNmAb005) [226]. The latter is particularly interesting because of recent successes with Aβ immunotherapies targeting soluble aggregates.

Although more than 300 AD clinical trials have been initiated [227,228], only two therapies have received USFDA approval over the past two decades—aducanumab [229] and lecanemab [230,231]—and both are conformational antibodies selective against soluble Aβ aggregates. On the other hand, active and passive immunotherapies against linear-sequence epitopes of Aβ have repeatedly failed in clinical trials [230,232]. The clinical success of lecanemab [233,234,235], the first disease-modifying AD therapy, lends support to the hypothesis that soluble aggregates are the underlying toxic agents in amyloid disorders. It also echoes neuropathological reports that high pathology controls—non-demented elderly with AD-like plaque and tangle burdens—have much lower levels of soluble Aβ oligomers and synaptic tau oligomers than AD patients [236]. These high-pathology control cases implicate that clearing soluble aggregates may be more important than clearing fibrils, highlighting an opportunity for developing immunotherapies against soluble tau aggregates. Thus far, the tau fibril-selective antibody Zagotenemab (MC-1) has failed in the phase 2 clinical trial, and the oligomer-selective antibody APNmAb005 is still in the phase 1 trial without available efficacy data. Whether conformational antibodies against soluble tau aggregates may be therapeutically effective remains to be determined. Because Aβ aggregates are mostly extracellular and tau aggregates are mostly intracellular, there has been some doubt on whether injected tau antibodies can find their targets after crossing the blood-brain barrier (at a rate of ~0.1%). The proposed mode of action for tau antibodies is to neutralize extracellularly released tau species [237,238]. There is also some evidence that a fraction of tau antibodies could enter neurons via endosomal pathways and co-localize with intracellular aggregates, possibly mediated by F_c_γ receptors [239,240,241]. Whether injected antibodies could neutralize tau species inside ectosomes, exosomes, or tunneling nanotubes remains to be investigated.

## 6. Conclusions and Future Directions

Although tau aggregation has been studied for almost four decades, the underlying molecular mechanisms remain elusive. Even at high concentrations under typical buffer conditions, wild-type tau proteins do not seem to self-coacervate into liquid droplets or self-assemble into oligomers or fibrils. This is consistent with the initial discovery of tau as a highly soluble, heat-stable protein. There have been many efforts to identify special conditions that induce wild-type tau aggregation. With molecular crowding, LLPS is observed, and oligomers may form upon droplet maturation but not fibrils. With hyperphosphorylation, self-assembly into oligomers is observed but not fibrils. With polyanionic cofactors such as heparin or RNA, it is possible to induce LLPS, oligomerization, and fibrillation.

In vivo, hyperphosphorylation appears to be the initiation event for aggregation because it dissociates tau from microtubules. It is often thought that tau aggregation is seeded extracellularly by GAGs and intracellularly by RNA. There is increasing evidence to suggest that soluble aggregates (oligomers and protofibrils) are the de facto toxic and seeding species in tauopathies, while fibrillar inclusions are relatively inert and represent secondary pathologies that appear much later. Although tau LLPS appears to occur within cells, whether it leads to oligomer or fibril formation remains to be determined.

One of the greatest obstacles in the study of oligomers and protofibrils is the lack of structural investigation tools. They are too heterogeneous to be analyzed as single particles under cryo-EM and too large to be effectively analyzed by solution NMR. The primary tools for investigating their structural features are conformational antibodies recognizing 3D epitopes. Even though cryo-EM has solved the atomic structure of core regions (covering MTBRs and R’) in many types of tau fibrils, the rest of the protein still resides within the “fuzzy coat”. Little is known about the structural roles of fuzzy-coat regions during oligomerization and fibrillation.

**Figure 6:**
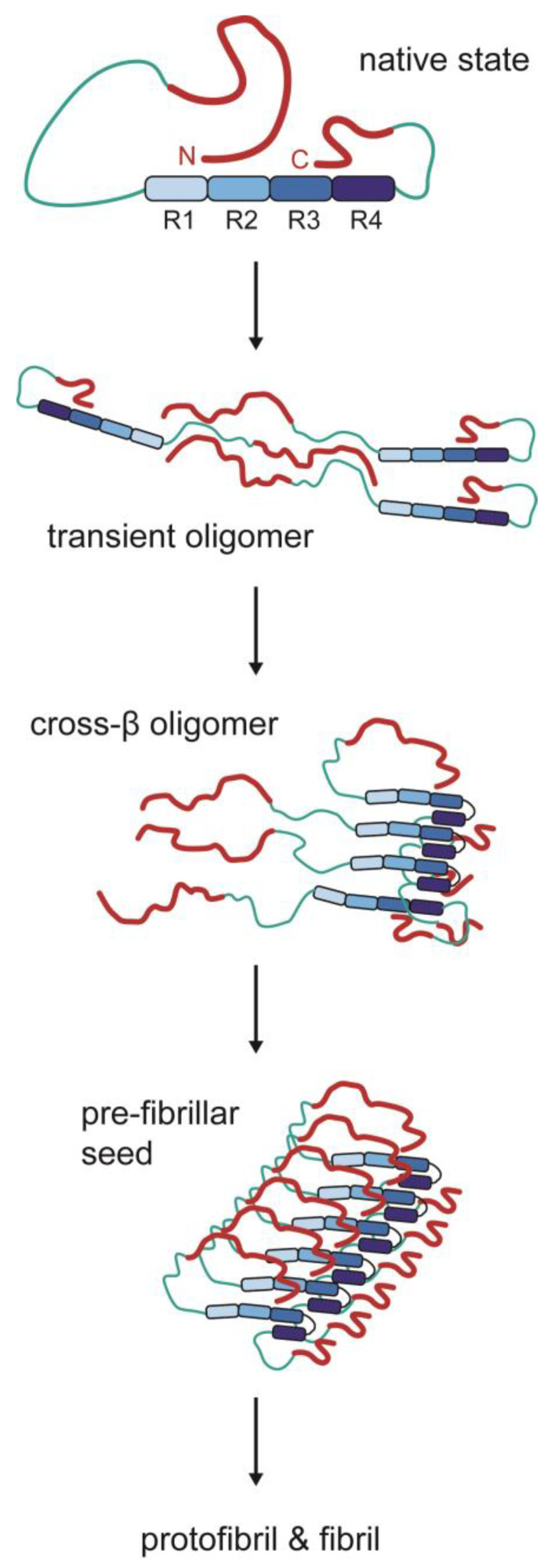
A hypothetical model of tau misfolding steps. (1) Native tau displays transient structural motifs, in which the N-terminus and C-terminus fold over MTBRs through electrostatic interactions (paperclip conformation). The negatively charged regions are marked as red ribbons, the positively charged regions as green ribbons, and the MTBRs (positively charged) as blue boxes. (2) The electrostatic interactions in the N-terminal half may mediate the formation of transient oligomers. (3) Within oligomeric assemblies, transient cross-β structures may gradually form around MTBRs. (4) The cross-β assemblies become more ordered as the extreme N-terminus becomes folded over MTBR3, forming prefibrillar seeds. (5) Normal tau proteins are recruited to prefibrillar seeds and converted into cross-β aggregates, eventually forming protofibrils and fibrils. There are no reported atomic structures for the fuzzy regions of tau marked as ribbons, so they are schematically presented as random coils. Please see the text for details.

Although tau is an IDP, there are still transient structural motifs in its native state, called the paperclip conformation (N-terminal and C-terminal regions attracted to the MTBD by electrostatic attraction). Based on the evidence discussed in previous sections, we may provide an “educated guess” about how different regions of tau participate in self-assembly. It seems that electrostatic interactions in the N-terminal half of the protein could drive phase separation and the formation of physiological and pathological oligomers. Hyperphosphorylation or polyanion binding could release the N-terminus from the MTBD and promote phase separation and oligomerization. Transient cross-β structures formed in the MTBR may contribute to droplet hardening or oligomer formation, and further evolution into stable cross-β structures eventually leads to protofibrils and fibrils. The initial step in fibril formation seems to involve folding the extreme N-terminus over R3, and the cross-β seeding region is the R3 hexapeptide (306–311). This tentative and hypothetical model, illustrated in Figure 6, will require further revisions as additional experimental evidence becomes available. The situation in the cellular milieu will be considerably more complex, where many tau proteoforms may have extensive interactions with other small molecules, macromolecules, and membrane structures. Because tau does not have a well-defined misfolding pathway toward a thermodynamically favored fibrillar state, these complex cellular factors will likely exert critical influences on the kinetic pathways of misfolding, which may explain why different tauopathies often exhibit diverse pathological features and fibril structures.

## Figures and Tables

**Table 2:** Conditions for inducing tau LLPS.

Tau Sequence	Tau Conc. (μM)	Buffer Condition	Inducer	Reference
Hi_6_-2N4R and His_6_-Δtau187 (255–441)	80	20 mM ammonium acetate, pH 7.0	poly(A) RNA or tRNA	Zhang et al., 2017 [197]
K18 (244–372)	100	50 mM sodium phosphate, pH 8.8	7.5% PEG	Ambadipudi et al., 2017 [198]
p-tau_2N4R_-EGFP-His_6_ (insect cell)	18–25	25 mM HEPES, 150 mM KCl, pH 7.4	10% dextran	Hernandez-Vega et al., 2017 [195]
p-tau_2N4R_, p-tau_1–256_, and p-tau_244–441_ (insect cells)	2–5	50 mM NaCl, pH 7.4	10% PEG or 12.5% Ficoll	Wegmann et al., 2018 [194]
p-tau oligomers from AD brains	n/a	50 mM NaCl, pH 7.4	10% PEG	Wegmann et al., 2018 [194]
tau_2N4R_ (A152T, ΔK280, P301L and P301S)	2	50 mM NaCl, pH 7.4	10% PEG	Wegmann et al., 2018 [194]
tau_2N4R_	2.5–20	5 mM sodium phosphate, pH 7.4	heparin or heparin-sepharose resin	Ferreon et al., 2018 [199]
tau_2N4R_	2–100	10 mM HEPES, 10–150 mM NaCl, pH 7.4	10–14%PEG	Boyko 2019 [193]
tau_2N4R_ (wt, P301L, G272V, and ΔK280)	5	10 mM HEPES, 100 mM NaCl	10% PEG	Boyko 2020 [200]
tau_2N4R_-GFP-His_6_ (wt, P301L, or S199E-S202E-T205E)	2	10 mM HEPES, 150 mM NaCl, pH 7.4	10% PEG	Kanaan et al., 2020 [201]
tau_2N4R_-His_6_ (FITC labeled)	2	10 mM HEPES, 100 mM NaCl, pH 7.6	75 µM arachidonic acid	Kanaan et al., 2020 [201]
His_6_-tau_255–441_ (C291S)	100	20 mM HEPES, pH 7.0	heparin, RNA, DNA, hyaluronan	Lin et al., 2020 [202]
His_6_-tau_2N4R_ (C291S-C322S)	20	20 mM sodium phosphate, pH 7.0	4.75 M NaCl	Lin et al., 2021 [196]
tau_2N4R_ (C291S-C322S)	10	5 mM NaCl, pH 7.0	none	Najafi et al., 2021 [203]
tau_0N4R_	5	10 mM HEPES, pH7.4	1 μM TIA1, 20 μg/mL RNA	Ash et al., 2021 [204]
tau_2N4R_ (C322S)	2	35 mM Tris, 150 mM NaCl pH 7.4	10% PEG	Foressi et al., 2023 [205]

**Table 3:** Tau immunotherapy clinical trials.

Antibodies	Therapy Type	Company	Isotype	Epitope	Clinical Trial	clinicaltrials.gov Identifier
AADvac1/ DC8E8	Active	Axon Neuroscience	N/A	294–305	1/2	NCT01850238—completed NCT02579252—completed NCT02031198—completed NCT03174886—unknown status
ACI-35/VAC20121/JNJ-64042056	Active	AC Immune	N/A	pS396/ pS404	2/3	NCT04445831—completed
APNmAb005	Passive	Aprinoia Therapeutics	IgG4	125–141; 153–157	1	NCT05344989—not recruiting
Bepranemab/UCB0107/UCB 0107/Antibody D	Passive	Roche	IgG4	235–250	1/2	NCT04658199—not recruiting NCT03464227—completed NCT03605082—completed NCT04185415—completed NCT04867616—not recruiting
BIIB076/NI-105	Passive	Biogen	IgG1	125–131	Discont.	NCT03056729—completed
BMS-986446/PRX005	Passive	Bristol-Myers Squibb	IgG1	MTBR (R1, R2, R3)	1	NCT06084598—not recruiting NCT06268886—not recruiting
E2814	Passive	Eisai	IgG1	299–303/ 362–366 (HVPGG)	1/2	NCT04231513—completed NCT04971733—recruiting NCT05269394—recruiting NCT01760005—recruiting
Gosuranemab/BIIB092/BMS-986168/IPN007	Passive	Biogen	IgG4	15–22	Discont.	NCT03352557—terminated NCT02460094—completed NCT03068468—terminated NCT03658135—terminated NCT02294851—completed NCT02658916—terminated
JNJ-63733657	Passive	Janssen	IgG1	pT212/pT217	2	NCT05407818—completed NCT04619420—not recruiting NCT03689153—completed NCT03375697—completed
Lu AF87908/hC10.2	Passive	Lundbeck	IgG1	pS396/pS404	1	NCT04149860—completed
MK-2214	Passive	Merck	N/A	pS413	1	NCT05466422—recruiting
PNT001	Passive	Pinteon	N/A	cis-pT231	1	NCT04096287—completed NCT04677829—terminated
RG7345/RO6926496	Passive	Roche	N/A	pS422	Discont.	NCT02281786—completed
Semorinemab/RO7105705/MTAU9937A/RG6100	Passive	AC Immune	IgG4	6–23	2	NCT02820896—completed NCT03828747—completed NCT03289143—terminated
Tilavonemab/ABBV-8E12/C2N 8E12/HJ8.5	Passive	AbbVie	IgG4	25–30	Discont.	NCT03712787—terminated NCT03413319—completed NCT03391765—terminated NCT02985879—terminated NCT02880956—completed
Zagotenemab/LY3303560	Passive	Eli Lilly	IgG1	7–9; 313–322	Discont.	NCT02754830—completed NCT03019536—completed NCT03518073—completed

## Data Availability

Not applicable.
